# Supplementary material for: The Propensity of the Human Liver to Form Large Lipid Droplets Is Associated with PNPLA3 Polymorphism, Reduced INSIG1 and NPC1L1 Expression and Increased Fibrogenetic Capacity
Source: Int J Mol Sci. 2021 Jun 5;22(11):6100. doi: 10.3390/ijms22116100 (PMC8200978; doi:10.3390/ijms22116100)
Supplement: Supplementary file 1 [file ijms-22-06100-s001.zip › ijms-1214263-supplementary.pdf]

Supplementary Table S1: donor demographics and clinical characteristics and liver graft percentage distribution of Ld-Mas and Sd-Mas calculated with respect to the total number of hepatocytes in the entire study population.

n	**225**
**Age (years)**	51.00 (34.00-64.00)
**Males, n (%)**	131 (58.2)
**BMI (kg/m2)**	24.84 (23.44-27.06)
**Diabetes, n (%)**	21 (9.3)
**Traumatic cause of death, n (%)**	73 (32.4)
**Noradrenaline administration, (%)**	115 (51.1)
**ICU stay (days)**	3.00 (2.00-6.00)
**Serum AST (IU/L)**	36.00 (25.00-67.00)
**Serum ALT (IU/L)**	31.00 (18.00-55.00)
**Liver graft Ld-MaS (percent of total hepatocytes) categorical, n (%):**	
**<5%**	110 (48.9)
**5-33%**	105 (46.7)
**34-66%**	10 (4.4)
**>66%**	0 (0)
**Liver graft Sd-MaS (percent of total hepatocytes) categorical, n (%):**	
**<5%**	153 (68.0)
**5-33%**	56 (24.9)
**34-66%**	13 (5.8)
**>66%**	3 (1.3)

Data are expressed as median and interquartile range or as numbers and proportions

Supplementary Table S2: liver graft mRNA expression of key enzymes involved in lipid metabolism, according to the presence and the predominant type of steatosis.

	**Nil steatosis**	**Pred. Sd-MaS**	**Pred. Ld-MaS**	**Overall P value**	**P value pred. Ld-Mas vs pred. Sd-Mas**	**P value pred. Ld-Mas vs nil steatosis**	**P value pred. Sd-Mas vs nil steatosis**
**Gene**	***n*=40**	***n*=15**	***n*=42**				
INSIG1	0.37 (0.08-2.24)	0.70 (0.19-1.91)	0.34 (0.13-0.99)	0.282	-	-	-
SREBP-2	1.58 (0.53-5.13)	1.42 (0.54-3.31)	0.89 (0.06-1.86)	**0.046**	0.329	0.057	1.000
HMGCR	3.12 (0.62-10.01)	2.28 (0.42-12.16)	0.67 (0.04-4.34)	0.052	-	-	-
NPC1L1	0.32 (0.08-1.92)	0.45 (0.08-1.51)	0.09 (0.00-0.30)	**0.001**	**0.011**	**0.003**	1.000
LDL-R	1.01	1.02	0.43	**0.049**	0.309	0.064	1.000

	(0.56-2.78)	(0.45-3.24)	(0.24-1.82)				
PCSK9	1.02 (0.38-5.28)	0.72 (0.11-2.49)	0.66 (0.32-3.16)	0.285	-	-	-
LXR	4.50 (2.12-7.99)	3.24 (1.15-9.41)	5.05 (2.45-10.72)	0.505	-	-	-
APOB	0.76 (0.20-2.82)	0.61 (0.26-1.10)	0.28 (0.10-1.02)	0.074	-	-	-
PNPLA3	3.58 (0.87-11.32)	2.31 (1.23-5.23	1.63 (0.46-4.41)	0.231	-	-	-

Data are expressed as median and interquartile range. Pred. Ld-MaS: predominant large droplet macrovescicular steatosis; Pred. Sd-MaS: predominant small droplet macrovescicular steatosis.
